# Endophthalmitis following Intravitreal Injection, Cataract Surgery, and Vitrectomy: Clinical Features and Visual Outcomes

**DOI:** 10.1155/2021/9985821

**Published:** 2021-09-20

**Authors:** Ana Maria Cunha, Maria Manuel Iglésias, Amândio Rocha-Sousa, Fernando Falcão-Reis, Manuel Falcão

**Affiliations:** ^1^Department of Ophthalmology, Centro Hospitalar Universitário de São João, Porto, Portugal; ^2^Faculty of Medicine, University of Porto, Porto, Portugal; ^3^Department of Surgery and Physiology, Faculty of Medicine of University of Porto, Porto, Portugal

## Abstract

**Purpose:**

To describe and compare the clinical features and visual outcomes of endophthalmitis following intravitreal injections (IVI), cataract surgery, and pars plana vitrectomy (PPV).

**Methods:**

This is a single-centre, retrospective study. All included patients had acute postoperative endophthalmitis secondary to one of these three procedures. Visual acuity (VA), comorbidities, time to presentation, and treatment were assessed. The primary outcome was visual outcome. A poor outcome was considered if final VA was worse than or equal to counting fingers (CF) and a good outcome was classified as VA better than CF.

**Results:**

Over 12 years, a total of 61 patients were included. Twenty-seven cases were post-cataract endophthalmitis; twenty-five were post-IVI and nine post-PPV. Endophthalmitis post-PPV had a worse visual outcome (88.9% of patients with VA worse than or equal to CF 95% CI 51.3 to 100.0%) than endophthalmitis following cataract surgery (25.9% of patients with VA worse than or equal to CF 95% CI 11.0 to 39.9%) and the IVI subgroup (44.0% of VA worse than or equal to CF 95% CI 24.0 to 67.0%) (*p*=0.001 and *p*=0.047). There were no significant differences in the proportion of patients with a poor visual outcome between endophthalmitis following cataract surgery and IVI (*p*=0.171).

**Conclusions:**

The number of patients with poor visual outcomes following acute endophthalmitis was similar in endophthalmitis following IVI and cataract surgery, but better than endophthalmitis following vitrectomy.

## 1. Introduction

Endophthalmitis is an infectious inflammation of the intraocular cavity. Although it is an uncommon disease, it is sight-threatening and may lead to irreversible vision loss [1]. Based on its type of transmission, it is possible to distinguish endogenous and exogenous causes, the latter being, by far, the most frequent [2]. Exogenous endophthalmitis is caused by direct inoculation via a penetrating ocular trauma or after any intraocular procedure [3]. Postoperative endophthalmitis (PE) can be classified as acute if it occurs within six weeks after surgery, and chronic, if it occurs after this period [4].

Cataract surgery is one of the most performed ophthalmologic surgeries in the developed world [4, 5]. Along with intravitreal injections (IVI), these are the two major causes of postoperative endophthalmitis [4, 5]. Endophthalmitis incidence following phacoemulsification and intraocular lens implantation is estimated to be between 0.012% and 1.3% since 2000 [4]. A large randomised clinical trial of the European Society of Cataract and Refractive Surgeons (ESCRS) compared the effect of intracameral injection of 1 mg of cefuroxime on the incidence of post-cataract endophthalmitis [6]. Cefuroxime prophylaxis significantly decreased the incidence of endophthalmitis from 0.3%–1.2% to 0.014–0.08% [6–11]. IVI of antivascular endothelial growth factor (anti-VEGF) agents such as bevacizumab, ranibizumab, and aflibercept are commonly used to treat neovascular age-related macular degeneration (AMD), diabetic macular edema (DME), and macular edema secondary to retinal vein occlusions. The estimated risk of post-anti-VEGF IVI endophthalmitis ranges from 0.008% to 0.06% per injection [12–14]. The exponential increase of intravitreal therapy in recent years has led to the rise in the total number of cases of endophthalmitis following IVI. The incidence rate of endophthalmitis following pars plana vitrectomy (PPV), with 20-gauge vitrectomy, ranged from 0.018% to 0.031% [15, 16]. With the more recent smaller incision vitrectomy kits (23- and 25-gauge), some studies have reported a higher PE rate with the incidence ranging from 0.03 to 0.14% [17–20].

Prevention, early detection, and adequate treatment are fundamental to the approach to this ophthalmologic emergency. Prevention should start before surgery by identifying the patient's risk factors. Several condition can increase the risk of PE endophthalmitis of both three surgical procedures. These conditions include blepharitis, conjunctivitis, lacrimal duct obstruction, palpebral malpositions, or previous operations on the cornea. The correction or treatment of these risk factors prior to surgery is desirable to reduce the risk of infection [21, 22].

Furthermore, prophylaxis with 5–10% povidone-iodine on the ocular surface has shown scientific evidence in reducing postoperative endophthalmitis [4]. In cataract surgery, intracameral cefuroxime also leads to significantly reduced PE rates [6]. Recent studies suggested that topical prophylaxis with antibiotics may be harmful and increase the risk of endophthalmitis following IVI [4, 19]. On the other hand, there is no conclusive evidence about topical antibiotics' effects in reducing endophthalmitis risk in cataract surgery and PPV [23].

PE endophthalmitis diagnosis depends mostly on clinical examination. It is based on findings of anterior chamber hypopyon and fibrin, ocular pain, corneal edema, inflammation, keratic precipitates, blurred vision, loss of red reflex secondary to vitreous opacification, and decreased visual acuity (VA) [3, 6, 24]. The diagnosis is considered a medical emergency requiring investigation and treatment within an hour of presentation.

Regarding treatment, IVI of antibiotics (vancomycin and ceftazidime) are the standard of emergency care [19, 24]. Recently, systemic adjuvant therapy with oral fourth-generation fluoroquinolones has been used [19, 25]. The role and timing of therapeutic PPV are still being discussed. Until now, the Endophthalmitis Vitrectomy Study (EVS) remains the only level I evidence randomised clinical trial that guides the main clinical protocols in treating endophthalmitis using intravitreal antibiotics and PPV. This study concluded that early vitrectomy in endophthalmitis was only beneficial in patients with vision of light perception or worse [19, 26]. However, diagnostic and therapeutic vitrectomy is the gold standard in the majority of cases [4, 19].

Postoperative endophthalmitis severity and prognosis depend on the virulence and inoculum of infecting bacteria, the patient's immune and visual status at presentation, the time from initial symptoms until the start of therapy, and the efficacy of treatment [4, 6, 26].

Although some studies compare postoperative endophthalmitis' clinical features following cataract surgery and IVI or cataract surgery and vitrectomy or IVI and vitrectomy [24, 27–30], in this study we compare clinical outcomes of these three procedures. The purpose of this study is to analyse and compare the clinical features and visual outcomes of endophthalmitis secondary to cataract surgery, post-IVI and post-PPV at a Portuguese tertiary referral hospital, Centro Hospitalar Universitário de São João (CHUSJ).

## 2. Methods

This retrospective cohort study examined patients diagnosed with acute endophthalmitis following cataract surgery, IVI, and PPV in the CHUSJ from February 2008 to February 2020. Within this period, all medical records of endophthalmitis in this hospital were reviewed. This study was conducted according to the Helsinki Declaration and was approved by the Ethics Committee of Centro Hospitalar Universitário de São João.

The inclusion criteria were patients who presented acute onset of symptoms and clinical findings consistent with endophthalmitis such as ocular pain, conjunctival injection, anterior chamber hypopyon and fibrin, loss of red reflex secondary to vitreous opacification, and vitreous opacities present on B-scan ultrasound. Patients included underwent cataract surgery, IVI, and PPV less than six weeks before presentation [4]. The ophthalmologic procedure was performed at CHUSJ or elsewhere, and patients were referred for care.

Clinical data were collected through review of electronic medical records as well as individual chart review. International Classification of Diseases 9^th^ and 10^th^ Edition (ICD-9 and ICD-10) codes for endophthalmitis were used to identify endophthalmitis cases electronically. All possible endophthalmitis cases from this electronic search were individually reviewed to confirm a diagnosis of presumed infectious endophthalmitis following cataract surgery, IVI, or PPV.

The exclusion criteria were endogenous or posttraumatic endophthalmitis, an uncertain diagnosis, more than six weeks following the surgical procedures, endophthalmitis caused by other surgical intervention, and missing data regarding final visual acuity.

### 2.1. Endophthalmitis Treatment Protocol

All patients included in this study began treatment as follows: in the emergency room, all patients underwent an intravitreous injection of 1 mg/0.1 ml of vancomycin and 2.25 mg/0.1 ml of ceftazidime at presentation. All patients were admitted to the hospital and received a systemic broad-spectrum antibiotic regimen for 10 days, including intravenous vancomycin 1 g every 12/12 h combined with ceftazidime, 1 g every 12/12 h oral prednisolone adjusted to the body weight (1 mg/kg until a maximum of 60 mg), and topical eye drops of dexamethasone (2/2 h). During the first 24 hours, patients were re-evaluated. Some patients were submitted to a three-port 23- or 25-gauge complete vitrectomy. The main indications were visual acuity of light perception or worse or no improvement within 24 hours of initial conservative therapy with intravitreal injections.

### 2.2. Outcomes

Our primary outcome measure was based on the final visual acuity and was dichotomised as good or poor visual outcome. Patients with a final VA of counting fingers (CF) or worse were included in the poor outcome. Based on final visual acuity, this dichotomisation is performed to overcome the bias created by the very different pathologies that lead to these three procedures. In fact, patients that perform intravitreal injections and vitrectomies have posterior segment disease, whereas patients that perform cataract surgery do not usually have concomitant retinal pathology.

Secondary outcomes collected included clinical features such as age, gender, comorbidities, therapeutic vitrectomy, time in days from surgical procedure to the onset of symptoms, presenting VA, time until the treatment of endophthalmitis with vitrectomy, final VA, and type of tamponade in PPV surgery.

### 2.3. Visual Outcome

For analysis, VA evaluated using the Snellen charts were converted to the logarithm of the minimum angle of resolution (logMAR) scale. According to Holladay, visual acuity equal to count fingers (CF) and hand motion (HM) correspond to logMAR + 2.0 and logMAR + 3.0, respectively [31]. Light perception is not actually a visual acuity measurement but merely a detection of stimulus and was assigned a logMAR of +4.0. No light perception (NLP) and cases of evisceration/enucleation were also classified as logMAR + 4.0. Presenting VA was measured at the presentation of endophthalmitis. Final VA was defined as the last follow-up visit.

### 2.4. Statistical Analysis

All statistical analyses were performed with SPSS® statistical software, version 26 (IBM SPSS Statistics for Windows, IBM Corporation, Armonk, NY). Statistical significance was considered at *p* < 0.05. The Kolmogorov–Smirnov test and normal probability plots were used to confirm the data's normal distribution. Data including age, time to the onset of symptoms, presenting VA, final VA, variation of VA, and time until vitrectomy followed a non-normal distribution, so it was assessed via Kruskal–Wallis Test followed by Dunn–Bonferroni post hoc tests. To analyse data such as visual outcome, gender, comorbidities and vitrectomy, we used the chi-squared test and for low count variables and Fisher's exact test.

## 3. Results

Over 12 years, from a total of 72 patients selected in our study, we excluded 11 patients due to missing data regarding final visual acuity. Therefore, in our study, we included 61 cases, with 27 cases being post-cataract patients, 25 post-IVI, and 9 post-PPV patients.

Baseline patient characteristics of the groups are presented in Table 1. The comparison of studied variables within the three subgroups is presented in Tables 1–4 . Twenty-five patients (41.0%) were male.

The median age of post-cataract endophthalmitis was 76.0 years (95% CI 71.9 to 78.4 years), 73.0 years for post-IVI endophthalmitis patients (95% CI 71.0 to 79.4 years), and 77.0 years for postvitrectomy (95% CI 70.0 to 81.4 years) (*p*=0.781). Median time to presentation was 6.0 days (95% CI 4.0 to 10.4 days) for post-cataract surgery, 4.0 days (95% CI 2.0 to 6.4 days) for post-IVI, and 3.5 days (95% CI 1.0 to 12.0 days) for post-PPV patients (*p*=0.259).

Median presenting visual acuity in the post-cataract group was +3.0 logMAR (range, 1.8–4.0 logMAR), in post-IVI was +3.0 logMAR (range, 2.0–3.8 logMAR), and in post-PPV +4.0 logMAR (range, 3.0–4.0 logMAR) (*p*=0.060).

From the forty-eight patients who underwent therapeutic vitrectomy, 17 were post-cataract patients (corresponding to 63.0% within this subgroup 95% CI 39.3% to 77.8%) while 23 were post-IVI patients (corresponding to 92.0% within this subgroup 95% CI 78.5% to 100.0%) (*p*=0.013).

The post-IVI patients waited 1.0 days (95% CI 1.0 to 2.0) until therapeutic vitrectomy and post-PPV group waited up to 2.5 days (95% CI 2.0 to 6.2 days) (*p*=0.042).

In terms of final VA, median final acuity in the post-cataract surgery group was +0.3 logMAR (range, 0.2–2.0 logMAR), +1.3 logMAR (range, 0.4–3.0 logMAR) in the IVI group, and, in the post-PPV, +4.0 logMAR (range, 3.5–4.0 logMAR) (*p*=0.002). However, only the differences between endophthalmitis post-cataract surgery and PPV were statistically different (*p*=0.002) whilst the difference between post-IVI and post-PPV almost reached statistically significance (*p*=0.066).

In terms of final visual outcomes, the cataract surgery subgroup had 25.9% of patients with a poor visual outcome (95% CI 11.0 to 39.9%) and the PPV subgroup had 88.9% of patients with a poor visual outcome (95% CI 51.3 to 100.0%) (*p*=0.001). The IVI subgroup had 44.0% of patients with poor visual outcome (95% CI 24.0 to 67.0%). When comparing post-IVI and PPV endophthalmitis, we found a significant statistical difference (*p*=0.047). There were no significant differences in the proportion of patients with a poor visual outcome between endophthalmitis following cataract surgery and IVI (*p*=0.171).

A subgroup analysis in the PPV subgroup was performed to identify the tamponade used in the initial vitrectomy. Silicon oil was used in 3 patients (33.3% 95% CI 11.1 to 66.7%) and gas in 4 patients (44.4% 95% CI 11.1 to 77.8%) as tamponade. In this analysis, we excluded 2 patients due to missing data.

Systemic comorbidities were present in 44.4% of post-cataract surgery (95% CI 25.9 to 71.8%), 80.0% of post-IVI (95% CI 56.0 to 96.0%), and 44.4% of post-PPV patients (95% CI 11.1 to 70.9%) (*p*=0.021). When we compared IVI and cataract surgery subgroup, patients with endophthalmitis following IVI had more systemic comorbidities than following cataract surgery (*p*=0.008).

The comparison of studied variables within the three subgroups is presented in Table 1, whereas the comparison within two of each of these procedures is presented in Tables 2–4.

Of these comparisons, we found a significant statistical difference between the group of post-cataract surgery and post-PPV in terms of final VA (*p*=0.002) and visual outcome (*p*=0.001). When we compared the patients with endophthalmitis post-PPV with the patients with endophthalmitis post-IVI, we only found significant statistical differences in time to vitrectomy (*p*=0.042) and visual outcome (*p*=0.047). Additionally, patients with endophthalmitis following IVI had more systemic comorbidities and were submitted more frequently to therapeutic vitrectomy (TV) than following cataract surgery (*p*=0.008 and *p*=0.013, respectively).

## 4. Discussion

This retrospective single-centre study at a Portuguese tertiary referral hospital compared 61 consecutive endophthalmitis cases following cataract surgery, IVI, and PPV. Cataract surgery was the most common surgical procedure, followed by IVI and PPV. These results were broadly consistent with previously published literature from other extensive, retrospective studies [28, 29].

Regarding the time from surgery to the onset of symptoms, the number of days was shorter for PPV (3.5 days), followed by IVI (4 days) and cataract surgery (6 days). These results from cataract surgery and IVI subgroups are consistent with recent data, where the mean time to presentation varies from 5 to 7.2 days from cataract surgery [24, 26, 29], 3 to 5.6 days after IVI [14, 24, 28, 29], and 3 to 8.8 days after PPV [15–18, 28, 32].

The median visual acuity at presentation was +3.0 logMAR (HM) in endophthalmitis post-cataract surgery, +3.0 logMAR (HM) in endophthalmitis following IVI, and +4.0 logMAR (LP) in endophthalmitis post-PPV. In IVI subgroup, our results are consistent with another study that described an average of presenting VA of 3.0 logMAR [33]. In post-cataract and PPV subgroups, we cannot directly compare to other similar studies as their authors use different visual acuity parameters to assess final vision acuity.

Moreover, in this study we report more cases of poor visual outcomes in endophthalmitis post-PPV as compared to post-cataract surgery and post-IVI injections with a statistically significant difference. The inferior visual outcome related to the endophthalmitis post-PPV compared to the other two procedures could be related to differences in the PPV procedure. Patients undergoing PPV usually have the vitreous cavity filled with a tamponade agent such as gas or silicone oil. The way that these tamponades interact with bacteria is largely unknown and they may potentiate their pathogenic properties [4, 19, 34]. On the other hand, the tamponade agents may slow the diffusion of intravitreal antibiotics, thus reducing their efficacy and leading to a worse prognosis. Finally, in our cohort, when comparing patients with direct bacterial inoculation of the posterior segment, PPV, and IVI, the time to vitrectomy of the latter group was shorter and this could have influenced the visual results. In our cohort, patients with endophthalmitis following vitrectomy were few. So, it was not possible to evaluate if different tamponades at the time of endophthalmitis diagnosis may influence the prognosis of the disease. Further studies must be performed to clarify this conclusion, especially in terms of the pathogenicity of different microorganism in each endophthalmitis.

Simunovic et al. showed that endophthalmitis following IVI has poorer visual outcomes (+2.0 logMAR [range +0.18 eNLP]) than the post-cataract subgroup (+0.40 logMAR [range +0.10 eNLP]) which was corroborated by many other studies [24, 28, 35]. These studies [24, 28] have shown that these poor outcomes in endophthalmitis following IVI were both associated with the fact that this infection had a higher prevalence of oral flora pathogens like *Streptococcus* spp. In our series, the final VA in the post-cataract group was better than in the post-IVI group, but the difference was not statistically significant.

However, because patients undergoing intravitreal injections and patients who perform vitrectomies have posterior segment disease, it would not be correct to compare patients' visual results based on final visual acuity. For example, patients who suffer endophthalmitis following cataract surgery do not usually have concomitant retinal pathology and have a higher chance of obtaining a better visual acuity than patients undergoing intravitreal injections due to wet AMD. As a result, to try and overcome this bias, we dichotomised patients based on final visual acuity dividing them into poor and good outcomes.

The proportion of patients with what we considered a favourable visual outcome was similar in the post-cataract and post-IVI group. Our study is more concordant with a different trend demonstrated in another study performed in the Sydney Eye Hospital, the same hospital of Simunovic et al.'s study [24] but performed more recently. (Simunovic et al.'s study was performed between 2007 and 2010 and Ong et al.'s study was performed between 2012 and 2017). The authors of this recent study [27] described a lack of statistically significant difference between both groups, which they justify by the change in causative infection organism, highlighting that *S. epidermidis* was the most frequent causative organism in endophthalmitis after post-IVI. Between these two studies performed in the same hospital, there was a modification in the protocol management of IVI, more precisely, the implementation of facemask and asking the patient to refrain from talking during the procedures. This alteration reduces aerosolisation droplet, explaining the change in flora and severity of endophthalmitis within this group of patients. So, our result can be explained by the aseptic protocol of IVI in this tertiary centre. As we do not have microorganism data included in this study, further studies must be performed.

Finally, our results also differ from the studies of Garg et al. [28], in which endophthalmitis after PPV had similar visual outcomes when compared with IVI and a similar meantime of clinical presentation (3.7 days versus 3.6 days).

There are limitations to this study. The first is due to its retrospective design which, which inevitably leads to heterogeneity of follow-up times, impossibility to control for confounding factors, and missing data. This last issue may be caused by a coding error or a misreported case that may underestimate endophthalmitis incidence. On the other hand, medical records have the advantage of assessing detailed chart patient review that allows us to confirm endophthalmitis' cases instead of relying on billing codes alone. The second limitation is the lack of analysis of preoperative information, intraoperative complication, and surgical time. Another specific limitation is that microbiological data was not available, so we cannot include it in our study. Despite a relatively larger number of endophthalmitis following intravitreal injections and cataract surgery for a very rare pathology, we can admit “small study effects,” particularly in the post-PPV group. Finally, our results can only be partially extrapolated for centres that use the same treatment protocols because we do not have data regarding microbiological etiology.

Despite these limitations, this study has several strengths. This study was performed in a single institution; no difference in the standardized preparation, institution, or even physician injection protocol interfered with differences in endophthalmitis caused by the three procedures. Moreover, endophthalmitis management was homogeneous, especially in terms of antibiotic IVI, systemic antibiotic regimen, and PPV.

In summary, our retrospective study of over 61 cases of endophthalmitis following cataract surgery, IVI, and PPV has two conclusions. First, the study found that the post-PPV subgroup is associated with significantly worse visual outcomes when compared with the post-cataract surgery and the IVI groups. However, we cannot state if the poorer outcome was because of the preexisting cause or because this disease was more virulent in the post-PPV group. Second, endophthalmitis following cataract surgery and IVI had similar visual outcomes when divided into poor or favourable outcomes.

## Figures and Tables

**Table 1:** Patient characteristic data of presumed endophthalmitis cases (*n* = 61) after cataract surgery, intravitreal injections (IVI), and pars plana vitrectomy (PPV) in Centro Hospitalar Universitário de São João.

	Total (*n* = 61)	Cataract surgery (*n* = 27)	PPV (*n* = 9)	IVI (*n* = 25)	*p* value
Age (y) [95% CI]	76,0 (69,0; 80,0) [73,0 to 78,0]	76,0 (70,0; 79,0) [71,9 to 78,4]	77,0 (65,0; 81,5) [70,0 to 81,4]	73,0 (65,0; 80,5) [71,0 to 79,4]	0,781

*Gender*					
Male [95% CI]	25 (41,0) [31,1 to 52,1]	11 (40,7) [25,9 to 55,6]	6 (66,7) [33,3 to 93,1]	8 (32,0) [16,0 to 48,0]	0,193
Female [95% CI]	36 (59,0) [47,9 to 68,9]	16 (59,3) [44,4 to 74,1]	3 (33,3) [6,9 to 66,7]	17 (68,0) [52,0 to 84,0]	

*Comorbidities*					
Yes [95% CI]	36 (59,0) [47,5 to 71,1]	12 (44,4) [25,9 to 71,8]	4 (44,4) [11,1 to 70,9]	20 (80,0) [56,0 to 96,0]	0,021
No [95% CI]	25 (41,0) [28,9 to 52,5]	15 (55,6) [28,2 to 74,1,7]	5 (55,6) [29,1 to 88,9]	5 (20,0) [4,0 to 44,0]	

*Vitrectomy*					
Yes [95% CI]	48 (78,7) [9,2 to 33,4]	17 (63,0) [39,3 to 77,8]	8 (88,9) [55,6 to 100,0]	23 (92,0) [78,5 to 100,0]	0,028
No [95% CI]	13 (21,3) [28,9 to 52,5]	10 (37,0) [22,2 to 60,7]	1 (11,1) [0,0 to 44,4]	2 (8,0) [0,0 to 21,5]	
Time to the onset of symptoms (days) [95% CI]	4,5 (2,0; 9,5) [3,0 to 4,0]	6,0 (3,0; 12,0) [4,0 to 10,4]	3,5 (1,5; 14,0) [1,0 to 12,0]	4,0 (2,0; 7,0) [2,0 to 6,4]	0,259
Presenting VA (logMAR) [95% CI]	3,0 (3,0; 4,0) [3,0 to 3,0]	3,0 (1,8; 4,0) [2,6 to 4,0]	4,0 (3,0; 4,0) [3,0 to 4,0]	3,0 (2,0; 3,8) [2,8 to 3,0]	0,060
Final VA (logMAR) [95% CI]	0,8 (0,3; 4,0) [0,4 to 2,0]	0,3 (0,2; 2,0) [0,2 to 0,9]	4,0 (3,5; 4,0) [3,6 to 4,9]	1,3(0,4; 3,0) [0,4 to 2,0]	0,002
Variation of VA (logMAR) [95% CI]	−0,7 (−2,7; 0,0) [−2,1 to −1,2]	−2,0 (−2,8; 0,0) [−2,7 to −0,4]	0,0 (−0,5; 1) [−2,00 to 1,0]	−1 (−2,0 0,0) [−1,8 to–0,9]	0,055
Time until vitrectomy (days) [95% CI]	2,00 (1,0; 3,0) [1,0 to 2,0]	2,0 (1,0; 3,5) [1,0 to 3,0]	2,5 (2,0; 6,0) [2,0 to 6,2]	1,0 (1,0; 2,0) [1,0 to 2,0]	0,035

*Visual outcome*					
Poor outcome, *n* (%) [95% CI]	26 (42,6) [26,2 to 59,6]	7 (25,9) [11,1 to 39,9]	8 (88,9) [51,3 to 100,0]	11 (44,0) [24,0 to 67,0]	0,004
Good outcome, *n* (%) [95% CI]	35 (57,4) [40,4 to 73,8]	20 (74,1) [60,1 to 88,9]	1 (11,1) [0,0 to 48,7]	14 (56,0) [33,0 to 76,0]	

Values are displayed as median (IQR) for continuous variables and number (%) for categorical variables. Comparisons were made with the chi-squared test and Fisher exact test for dichotomous data. For continuous variable, nonparametric Kruskal–Wallis test was used. The level of statistical significance was set at *p* < .05. Poor outcome was classified as VA worse than or equal than counting fingers (CF) and good outcome classified as VA better than CF. PPV = pars plana vitrectomy; IV = intravitreal injection; VA = visual acuity; logMAR = logarithm of the minimal angle of resolution.

**Table 2:** Variable comparison and analysis of presumed endophthalmitis cases (*n* = 61) after cataract surgery and pars plana vitrectomy (PPV) in Centro Hospitalar Universitário de São João.

	Cataract surgery (*n* = 27)	PPV (*n* = 9)	*p* value
Final VA (logMAR) [95% CI]	0,3 (0,2; 2,0) [0,2 to 0,9]	4,0 (3,5; 4,0) [3,6 to 4,9]	0,002
Time until vitrectomy (days) [95% CI]	2,0 (1,0; 3,5) [1,0 to 3,0]	2,5 (2,0; 6,0) [2,0 to 6,2]	0,739

*Vitrectomy*			
Yes [95% CI]	17 (63,0) [39,3 to 77,8]	8 (88,9) [55,6 to 100,0]	0,223
No [95% CI]	10 (37,0) [22,2 to 60,7]	1 (11,1) [0,0 to 44,4]	

*Comorbidities*			
Yes [95% CI]	12 (44,4) [25,9 to 71,8]	4 (44,4) [11,1 to 70,9]	1,000
No [95% CI]	15 (55,6) [28,2 to 74,1,7]	5 (55,6) [29,1 to 88,9]	

*Visual outcome*			
Poor outcome, *n* (%) [95% CI]	7 (25,9) [11,1 to 39,9]	8 (88,9) [51,3 to 100,0]	0,001
Good outcome, *n* (%) [95% CI]	20 (74,1) [60,1 to 88,9]	1 (11,1) [0,0 to 48,7]	

Values are displayed as median (IQR) for continuous variables and number (%) for categorical variables. Comparisons were made with nonparametric Kruskal–Wallis test following Dunn-Bonferroni post hoc tests. The level of statistical significance was set at *p* < .05. Poor outcome was classified as VA worse than or equal than counting fingers (CF) and good outcome classified as VA better than CF. PPV = pars plana vitrectomy; VA = visual acuity; logMAR = logarithm of the minimal angle of resolution.

**Table 3:** Variable comparison and analysis of presumed endophthalmitis cases (*n* = 61) after intravitreal injections (IVI) and pars plana vitrectomy (PPV) in Centro Hospitalar Universitário de São João.

	IVI (*n* = 25)	PPV (*n* = 9)	*p* value
Final VA (logMAR) [95% CI]	1,3(0,4; 3,0) [0,4 to 2,0]	4,0 (3,5; 4,0) [3,6 to 4,9]	0,066
Time until vitrectomy (days) [95% CI]	1,0 (1,0; 2,0) [1,0 to 2,0]	2,5 (2,0; 6,0) [2,0 to 6,2]	**0,042**

*Vitrectomy*			
Yes [95% CI]	23 (92,0) [78,5 to 100,0]	8 (88,9) [55,6 to 100,0]	**1,000**
No [95% CI]	2 (8,0) [0,0 to 21,5]	1 (11,1) [0,0 to 44,4]	

*Comorbidities*			
Yes [95% CI]	20 (80,0) [56,0 to 96,0]	4 (44,4) [11,1 to 70,9]	**0,085**
No [95% CI]	5 (20,0) [4,0 to 44,0]	5 (55,6) [29,1 to 88,9]	

*Visual outcome*			
Poor outcome, *n* (%) [95% CI]	11 (44,0) [24,0 to 67,0]	8 (88,9) [51,3 to 100,0]	**0,047**
Good outcome, *n* (%) [95% CI]	14 (56,0) [33,0 to 76,0]	1 (11,1) [0,0 to 48,7]	

Values are displayed as median (IQR) for continuous variables and number (%) for categorical variables. Comparisons were made with nonparametric Kruskal–Wallis test following Dunn–Bonferroni post hoc test. The level of statistical significance was set at *p* < .05. Poor outcome was classified as VA worse than or equal than counting fingers (CF) and good outcome classified as VA better than CF. PPV = pars plana vitrectomy; IVI = intravitreal injection; VA = visual acuity; logMAR = logarithm of the minimal angle of resolution.

**Table 4:** Variable comparison and analysis of presumed endophthalmitis cases (*n* = 61) after cataract surgery and intravitreal injections (IVI) in Centro Hospitalar Universitário de São João.

	Cataract surgery (*n* = 27)	IVI (*n* = 25)	*p* value
Final VA (logMAR) [95% CI]	0,3 (0,2; 2,0) [0,2 to 0,9]	1,3(0,4; 3,0) [0,4 to 2,0]	0,336
Time until vitrectomy (days) [95% CI]	2,0 (1,0; 3,5) [1,0 to 3,0]	1,0 (1,0; 2,0) [1,0 to 2,0]	0,332

*Vitrectomy*			
Yes [95% CI]	17 (63,0) [39,3 to 77,8]	23 (92,0) [78,5 to 100,0]	**0,013**
No [95% CI]	10 (37,0) [22,2 to 60,7]	2 (8,0) [0,0 to 21,5]	

*Comorbidities*			
Yes [95% CI]	12 (44,4) [25,9 to 71,8]	20 (80,0) [56,0 to 96,0]	**0,008**
No [95% CI]	15 (55,6) [28,2 to 74,1,7]	5 (20,0) [4,0 to 44,0]	

*Visual outcome*			
Poor outcome, *n* (%) [95% CI]	7 (25,9) [11,1 to 39,9]	11 (44,0) [24,0 to 67,0]	0,171
Good outcome, *n* (%) [95% CI]	20 (74,1) [60,1 to 88,9]	14 (56,0) [33,0 to 76,0]	

Values are displayed as median (IQR) for continuous variables and number (%) for categorical variables. Comparisons were made with nonparametric Kruskal–Wallis test following Dunn–Bonferroni post hoc tests. The level of statistical significance was set at *p* < .05. Poor outcome was classified as VA worse than or equal than counting fingers (CF) and good outcome classified as VA better than CF. PPV = pars plana vitrectomy; IVI = intravitreal injection; VA = visual acuity; logMAR = logarithm of the minimal angle of resolution.

## Data Availability

The data used to support the findings of this study are available from the corresponding author upon request.
